# Essential emergency and critical care as a health system response to critical illness and the COVID19 pandemic: what does it cost?

**DOI:** 10.1186/s12962-023-00425-z

**Published:** 2023-02-13

**Authors:** Lorna Guinness, Angela Kairu, August Kuwawenaruwa, Karima Khalid, Khamis Awadh, Vincent Were, Edwine Barasa, Hiral Shah, Peter Baker, Carl Otto Schell, Tim Baker

**Affiliations:** 1 Center for Global Development, Great Peter House, Abbey Gardens, Great College St, London, SW1P 3SE UK; 2 Global Health Economics Centre, London School of Hygiene and Tropical Medicine, London, UK; 3 Health Economics Research Unit, KEMRI Wellcome Trust Research Programme, Nairobi, Kenya; 4 Ifakara Health Institute, Dar es Salaam, Tanzania; 5 Muhimbili University of Health and Allied Sciences, Dar Es Salaam, United Republic of Tanzania; 6 Centre for Tropical Medicine and Global Health, Nuffield Department of Medicine, University of Oxford, Oxford, UK; 7 Department of Global Public Health, Karolinska Institutet, Stockholm, Sweden; 8 Centre for Clinical Research Sörmland, Uppsala University, Eskilstuna, Sweden; 9 Department of Medicine, Nyköping Hospital, Nyköping, Sweden; 10 Department of Clinical Research, London School of Hygiene & Tropical Medicine, London, UK

**Keywords:** Critical care, COVID-19, Cost, Kenya, Tanzania, Sub-Saharan Africa, Essential emergency critical care

## Abstract

Essential Emergency and Critical Care (EECC) is a novel approach to the care of critically ill patients, focusing on first-tier, effective, low-cost, life-saving care and designed to be feasible even in low-resourced and low-staffed settings. This is distinct from advanced critical care, usually conducted in ICUs with specialised staff, facilities and technologies. This paper estimates the incremental cost of EECC and advanced critical care for the planning of care for critically ill patients in Tanzania and Kenya.

The incremental costing took a health systems perspective. A normative approach based on the ingredients defined through the recently published global consensus on EECC was used. The setting was a district hospital in which the patient is provided with the definitive care typically provided at that level for their condition. Quantification of resource use was based on COVID-19 as a tracer condition using clinical expertise. Local prices were used where available, and all costs were converted to USD2020.

The costs per patient day of EECC is estimated to be 1 USD, 11 USD and 33 USD in Tanzania and 2 USD, 14 USD and 37 USD in Kenya, for moderate, severe and critical COVID-19 patients respectively. The cost per patient day of advanced critical care is estimated to be 13 USD and 294 USD in Tanzania and USD 17 USD and 345 USD in Kenya for severe and critical COVID-19 patients, respectively.

EECC is a novel approach for providing the essential care to all critically ill patients. The low costs and lower tech approach inherent in delivering EECC mean that EECC could be provided to many and suggests that prioritizing EECC over ACC may be a rational approach when resources are limited.

**Graphical Abstract:**

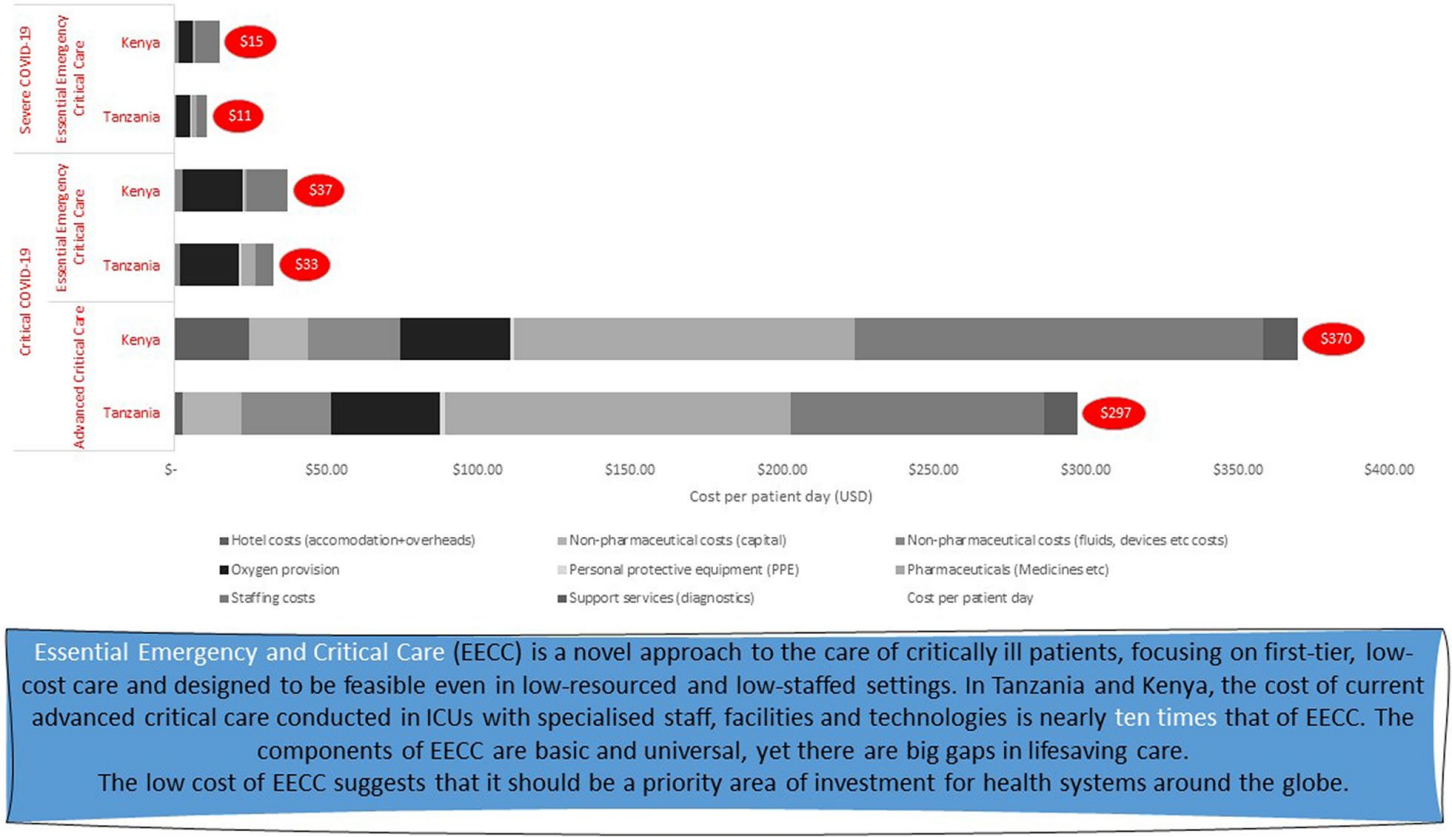

**Supplementary Information:**

The online version contains supplementary material available at 10.1186/s12962-023-00425-z.

## Background

**Fig. 1:**
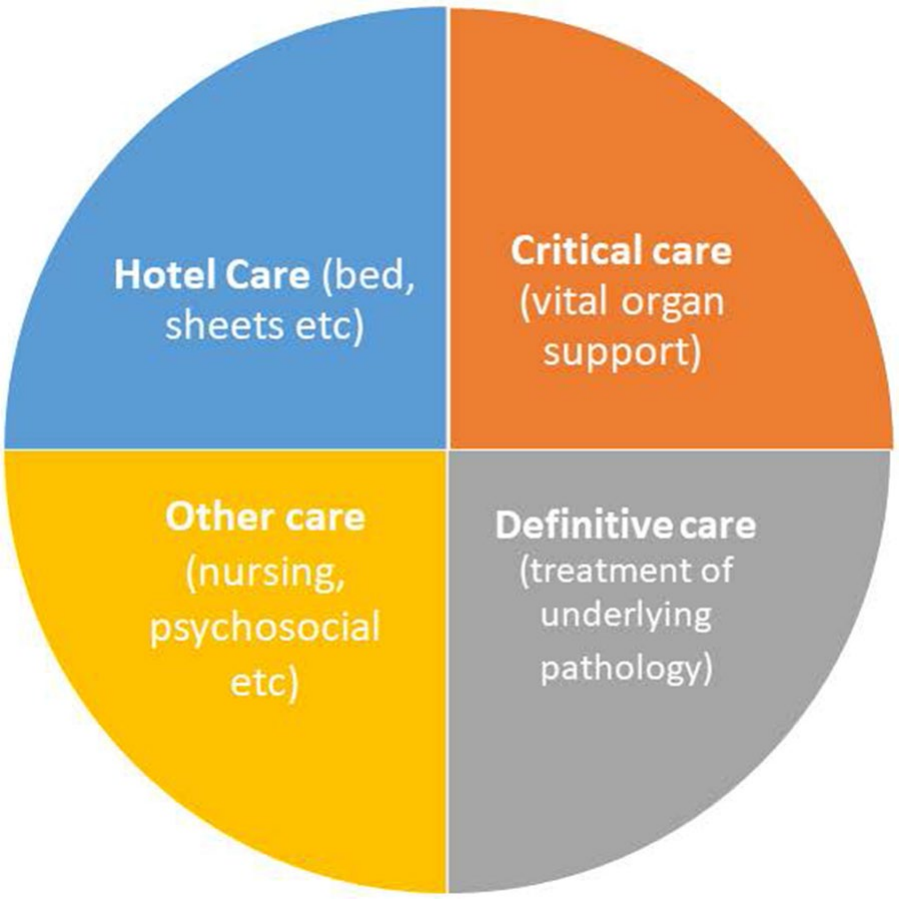
Domains of care

Globally, the burden of critical illness is higher and most deaths from critical illness occur in Low- and Middle- Income Countries (LMICs) [1, 2]. Effective treatment of the patients with the highest risk of death has the potential to save many lives. Yet a large unmet need of even basic care of critical illness has been reported from hospitals in Africa [3–6]. Critical care can be seen as the supportive care that keeps a patient’s vital organs functioning and is distinct from the definitive care provided for the underlying pathology (Fig. 1). Improving access to care for critical illness in resource poor settings is a challenge. Increasing the availability of advanced critical care (ACC), including mechanical ventilation, is constrained by a broader lack of health system resources and capacity [7]. Scaling up critical illness care that presents value for money needs to acknowledge unmet needs at all levels from essential to advanced critical illness care, capacity constraints such as shortages of human resources and a lack of maintained and functioning equipment [1, 8–11]. One solution is to ensure that life-saving, essential treatments for critical illness is available for all critically ill patients, an approach that underpins the Essential Emergency and Critical Care (EECC) model [12–14].

EECC was originally developed as a global concept [13], a horizontal approach to care focusing on the first-tier low-cost care of critical illness and designed to be feasible even in low-resourced and low-staffed settings [13]. Since then an international Delphi consensus has generated the full content of EECC [12]. EECC is distinct from ACC which includes the resource-intensive care of critical illness such as mechanical ventilation and other organ support, usually conducted in Intensive Care Units (ICUs) with specialised staff, facilities and technologies. The EECC approach has received increasing attention during the COVID-19 pandemic, which has been a critical illness crisis. As the pandemic took hold, critical illness care—support for patients’ vital organ functions—became the primary therapeutic means for reducing mortality. With scale-up and effective use of critical illness care seen as a fundamental determinant of the impact of the pandemic in many settings [14], the pandemic highlighted the shortage of capacity and resources for such care [8, 9, 15–17]. These resource constraints lead to poor health outcomes. Even prior to the pandemic, mortality in hospitalised patients in Africa was significantly higher than the global average [18]. Donors and policy-makers were therefore seeking out new solutions to the improvement of care for COVID-19 patients [2, 5, 14].

While it is generally reported that critical care is expensive, the costs of providing care for the critically ill is poorly understood [2, 19]. Heterogeneity of patients, treatment and locality of treatment make costing exercises and economic evaluation challenging and costly [19]. A further obstacle to economic analysis is in identifying the supportive care that is specific for critical illness while terminologies and definitions are not used consistently [2, 20]. Resource poor settings where capacity and budgets are particularly constrained need evidence on costs of different approaches to critical illness care to plan and allocate their budgets. For example, a recent review of the costs of critical care in Tanzania found no data available data to determine these costs versus the definitive care [21] and a systematic review of cost-effectiveness analyses in ICUs found incremental cost-effectiveness ratios relating to a broad range of strategies including treatment of sepsis and acute respiratory failure as well as “general intensive care interventions”[19]. Similarly, while critical care costs have been identified as part of the effort to cost COVID-19 care in Kenya [22], further cost information is still needed to determine the affordability and relative efficiency of critical care strategies, including EECC, as well as inform budgeting across different settings.

Tanzania is a lower-middle income country in East Africa with a population of 63 million [23]. Availability of ICUs is limited with a total of 38 ICU beds across the four national referral hospitals and a scarcity of resources available to provide critical care [10, 24, 25]. Kenya, also in East Africa and a lower-middle income country, has a population of 53 million [23]. A recent survey found that oxygen therapy was only available in 78% of facilities, that there were 537 ICU beds and, as with Tanzania, varied availability of the resources required for delivery of effective critical illness care [26]. The limited availability of critical illness care in these settings suggests that both countries should consider the introduction of EECC. Before rolling out EECC, it is important to estimate the budgetary implications of providing this care. This paper generates estimates of the cost per patient day of EECC and ACC in Tanzania and Kenya using the process of a normative incremental costing, from a healthcare systems perspective, in order to capture the total direct costs of treatment.

## Methods

### Study setting

EECC and ACC were costed for two settings: Tanzania and Kenya, two lower-middle income countries in East Africa (see Table 1). In Tanzania the healthcare referral system is organized in a pyramid structure, with the community centre at the base, followed by dispensary, health centres and district hospitals that constitute primary health care. These are followed by regional referral hospitals, zonal hospitals and specialized or national hospitals [27, 28]. Facility surveys have found capacity for critical care to be limited, particularly at the district hospital level [10, 29]. In Kenya, the health system is also decentralised with resource allocation decisions devolved to the county level. Health facilities are classified into levels 2 to 6, with level 2 including health centres and dispensaries and level 6 facilities provide all specialist services. Like Tanzania, the system faces challenges in availability of resources including consumables, equipment and human resources at the facility level as well as regional disparities in availability of care with 22 out of 47 counties having at least one intensive care unit [16, 30, 31]. We assume a setting where constant electricity supply is not guaranteed, to reflect the common black outs in both Tanzania and Kenya, but that care is being provided in a hospital, the patient has a bed, the patient is provided with the definitive aspects of care (see Fig. 1) and daily nutritional needs unless this forms part of critical illness care, e.g. parenteral feed, the facility is cleaned and running water is available.

**Table 1:** Country characteristics

	United Republic of Tanzania	Kenya	Source
Population	63.5 million	53.0 million	[23]
GDP per capita (Int$)	2836	5211	[23]
Healthcare expenditure (% of GDP)	3.83	4.59	[23]
Infant mortality rate (per 1000 live births)	35	31	[23]
ICU beds	38^a^	537	Tanzania: [24] Kenya: [26]

^a^across the four national referral hospitals

### Intervention description

Critical care is the support of vital organ functions and complements the other care provided to a patient: the definitive care for the patient’s pathology (e.g. curative therapies such as drugs and surgery), hotel care (beds, sheets etc.) and other care (e.g. psychosocial support) (see Fig. 1). To provide effective coverage of EECC there are two core streams of activity: identification of the critically ill; and essential treatment of the critically ill [13]. The identification stream involves the processes for identifying critical illness, distinct from the diagnosis of the underlying condition, and requires the monitoring of vital signs (body temperature, pulse rate, respiration rate, blood pressure). The treatment stream is the organ support for a critically ill patient, including oxygen therapy, intravenous fluid resuscitation, and maintaining a free airway through correct positioning of unconscious patients.

ACC was defined as the provision of EECC combined with the advanced resource-intensive care of critical illness typical of an ICU. To specify the content of ACC over and above EECC resources, we documented the current facilities and practices in the best resourced ICUs in Tanzania—care that includes mechanical ventilation and other advanced organ support, conducted with specialised staff, facilities and technologies. The team specifying the content of ACC comprised critical care physicians with experience of critical care, including COVID-19, globally as well as specific experience of the Tanzanian and Kenyan health systems.

### Approach to costing

#### Identifying resources

The EECC Delphi consensus [12], was used to define all inputs required to provide EECC over and above the other care provided in the hospital setting (see Fig. 1) and a list of inputs for ACC, excluding those required for EECC, was developed by the research team based on the intervention definition of advanced critical care (see Additional file 1). Both lists involved a set of equipment, consumables, drugs, human resources with staff trained to deliver care according to specific routines, and guidelines. Costs were classified into the categories of Human Resources, Consumables (Pharmaceutical and non-pharmaceutical), Oxygen, Personal Protective Equipment (PPE) and Equipment. In costing EECC, we assumed a similar level of health facilities as a typical district hospital in Tanzania and a level 4 county hospital in Kenya. These characterizations informed the assumptions made around the specification of resource items.

Oxygen therapy is a core component of critical care for patients with COVID-19, as well as other conditions, and is a major portion of the cost [22, 32]. Oxygen therapy can be provided in different ways, depending on the needs of the patient and the setting or access to resources. We specified two different oxygen scenarios for EECC in the district hospital setting: (1) oxygen concentrators with cylinders as back up; (2) oxygen cylinders; and (3) one further scenario for higher flow oxygen in the ICU for ACC.

#### Quantifying resource use

Care for the critically ill varies according to the patient’s diagnosis and the severity of their critical illness. This heterogeneity means that an accurate quantification of resource use for all critically ill patients would require a large sample of patient observations. In the absence of such data, we selected COVID-19 as a tracer condition. COVID-19 provides a unique opportunity to monitor a large sample of critically ill patients with a degree of homogeneity. COVID-19 is likely to be most representative of critical illness with respiratory failure and due to infections so that the generalisability of costs to other types of critical illness will be more limited. We estimated treatment costs for COVID-19 patients in three separate severity categories—moderate, severe, and critical, based on WHO definitions described in the "Living guidance for clinical management of COVID-19" (https://www.who.int/publications/i/item/WHO-2019-nCoV-clinical-2021-2, accssed Feb 2021) . According to these definitions, patients with both severe and critical COVID-19 have vital organ dysfunctions and are therefore “critically ill” [34]. Patients with moderate COVID-19 are not critically ill and so resources are only used within the identification stream for EECC to identify those who develop severe COVID-19 and are not included in the ACC costing. For patients with severe COVID-19, resources are used within the identification and treatment streams of EECC and for ACC they are assumed to receive treatment as for EECC plus additional resources in the identification stream to identify those who develop critical COVID-19. For patients with critical COVID-19, resources are used across the identification and treatment streams for both EECC and ACC.

Resource use of the items specified in the input categories was quantified based on expert opinion. Clinicians on the research team, experienced in care of critically ill COVID patients, were asked to estimate the average resource use per patient per day of each included item for patients with moderate, severe and critical COVID-19. Two physicians provided estimates independently, the estimates were compared, and the physicians then discussed and resolved their differences to generate an agreed estimate.

#### Valuing resource use

All resources were costed at current (2020) prices to generate economic costs. Where possible local prices were sought. Capital costs were annualized using a discount rate of 3% as recommended in international guidance [35]. All costs were converted to USD where necessary using current exchange rates (1 USD = 2300 TZS, 115 KSh) [36, 37].

In both countries, public sector staff salaries data were used to value staff time. The key source of prices for consumables and equipment in Tanzania were the Tanzanian Government’s Medical Supplies Department. Where shipping costs were excluded from prices, we included a 17.4% uplift to account for transportation and insurance as recommended by the Medical Supplies Department. In Kenya, sources included the Kenya drug index catalogue and a 2018 survey of 20 healthcare facilities [38]. Where prices were not available locally, we obtained costs from the UNICEF supply procurement list, South African medical supplies price list, experts in critical care in sub-Saharan Africa and, finally, internet searches (see Additional file 2). Hospital hotel costs were based on daily charge rates for both countries.

The PATH oxygen and costing tool provides a means of estimating the cost per litre per minute of oxygen under different scenarios for different settings (https://www.path.org/resources/quantification-and-costing-tools/). To generate a cost per litre of oxygen using the tool, we were required to provide data on hospital characteristics—including number and type of beds and bed occupancy. We used a scenario for a typical district hospital in Tanzania based on hospital facility surveys [10, 39]. The PATH costs include transportation and logistics for the oxygen supply as well as back up supply and the power costs and captures both capital and recurrent costs. We complemented this with local prices collected in 2021 (the price for a cylinder refill) (see Additional file 3). No equivalent data were available for Kenya at the time of the study and therefore we used the Tanzania oxygen cost estimates for both countries.

#### Generating unit costs and scenario analysis

A cost per patient day was derived for a reference scenario based on the resource use per patient day for each category of patient for EECC and ACC. For the reference cost scenario, the costs are defined as the costs of EECC resources that need to be in place, without which EECC could not be provided.

Ranges for the cost per patient day were developed based on different scenarios that address uncertainties in the key assumptions for oxygen and staffing. In the case of oxygen, the cost of oxygen and oxygen supplies were increased and decreased by an indicative 25%, to illustrate the potential impact of the fluctuation in prices during the pandemic. In the case of staffing, for a low cost estimate we identified the lowest cost staff that could carry out the tasks from the staff list staff salary costs; for the high cost estimate staff salary costs used were at the highest grade for nurses and a consultant level salary used for the doctor. For ACC, we further varied pharmaceutical costs by increasing and decreasing their total cost per day by 25% to demonstrate the degree to variations in the drug cost influence on total cost. Finally we used the upper and lower bounds of cost generated in the scenario analyses to carry out a probabilistic analysis. For each broad category of input, a Monte Carlo simulation (n = 1000) based on a gamma distribution was implemented to generate a mean and confidence interval for the cost per patient day for EECC and ACC in both countries [40].

An additional analysis was carried out to generate the cost per patient by multiplying the cost per patient day by the average length of stay for different patient categories. The evidence on length of stay for COVID-19 patients in the different categories of severity was obtained from a systematic review of the international literature [41]. As length of stay for the different categories of patient were not readily available for the Tanzanian and Kenya contexts, we used the most robust source of data from literature which was from the United Kingdom.

## Results

### Costs of critical care strategies in Tanzania and Kenya

The costs of EECC and ACC are presented in Tables 2 and 3. The cost per patient day of EECC in Tanzania is estimated to be 1 USD, 11 USD and 33 USD for patients with moderate (identification only), severe and critical COVID-19 respectively. For moderate patients, staff time for checking vital signs to identify critical illness is the costliest input (85% of the total) while Personal Protective Equipment (PPE) makes up 12% of costs. For severe and critical patients, the cost of oxygen provision is the most important cost (44% and 60%, respectively), with staff time being a relatively smaller proportion of the total (31% and 19% respectively). Pharmaceuticals are 16% and 14% of total costs for severe and critical patients; and as severity increases PPE is a relatively less important contributor to costs (2.2% and 1.6% of costs for severe and critical patients).

**Table 2:** Incremental costs per patient day of Essential Emergency Critical Care for patients with moderate, severe and critical COVID-19 in Tanzania and Kenya, USD (2020 prices)

	Tanzania						Kenya					
	Moderate		Severe		Critical		Moderate		Severe		Critical	
	USD	%	USD	%	USD	%	USD	%	USD	%	USD	%
Hotel costs (accommodation + overheads)	0.01	1	0.01	0.1	0.01	0.04	0.06	3.56	0.08	0.5	0.09	0.2
Staffing costs	0.86	85	3.35	31	6.11	19	1.54	87.65	8.01	54	13.55	36
Oxygen provision			4.74	44	19.67	60	–	0.00	4.74	32	19.68	53
Pharmaceuticals (Medicines etc.)			1.70	16	4.71	14	–	0.00	0.54	4	0.72	2
Non-pharmaceutical costs (capital)	0.02	2	0.13	1	0.15	0.5	0.02	1.38	0.15	1	0.15	0.4
Non-pharmaceutical costs (fluids, devices etc. costs)	0.00	0	0.66	6	1.62	5	–	0.00	1.16	8	2.73	7
Personal protective equipment (PPE)	0.12	12	0.24	2	0.57	2	0.13	7.41	0.18	1	0.52	1
Patient cost/day USD^b^	1.02 (0.33–2.25)		10.74 (6.25–17.98)		32.77 (25.31–44.75)		1.76 (1.18–2.56)		14.32 (11.18–18.42)		36.96 (29.80–45.68)	
Cost per patient per stay in hospital^a^	3.03	100	86.64	100	262.75	100	5.33	100	118.86	100	524.06	100

^a^Percentages may not add to 100 due to rounding

^b^Mean and confidence interval generated using a Monte Carlo simulation (n = 1000) based on a gamma distribution with limiting values on the input cost categories defined by the scenario analyses

**Table 3:** Incremental costs per patient day of Advanced Critical Care for patients with severe and critical COVID-19 in Tanzania and Kenya, USD (2020 prices)

	Tanzania				Kenya			
	Severe		Critical		Severe		Critical	
	USD	%	USD	%	USD	%	USD	%
Hotel costs (accommodation + overheads)	0.01	0.1	2.90	1	0.08	0.4	24.73	10
Staffing costs	5.63	43	83.72	28	10.48	60	134.19	6
Oxygen provision	4.74	36	36.17	12	4.74	27	36.17	14
Pharmaceuticals (Medicines etc.)	1.70	13	113.40	38	0.54	3	112.46	45
Non-pharmaceutical costs (capital)	0.13	1	19.45	7	0.15	0.8	19.45	8
Non-pharmaceutical costs (fluids, devices etc. costs)	0.66	5	29.15	10	1.16	7	30.25	12
Personal protective equipment (PPE)	0.24	2	1.68	0.6	0.18	1	1.14	0.5
Support services (diagnostics)	0.00	0	10.83	4	0.00	0	11.25	4
Patient cost/day USD^b^	13.11 (9.20–18.29)		293.77 (250.37–344.88)		17.20 (15.30–19.17)		344.85 (275.25–430.29)	
TOTAL Cost per patient per stay in hospital^a^	104.87	100.00	4162.18	100.00	242.56	100	5174.98	100.00

^a^Percentages may not add to 100 due to rounding

^b^Mean and confidence interval generated using a Monte Carlo simulation (n = 1000) based on a gamma distribution with limiting values on the input cost categories defined by the scenario analyses

The cost per patient day of ACC is estimated to be 13USD for severe and 294USD for critical patients (see table 3). For patients in the critical category, pharmaceuticals are the most important cost category (38%) with staff and oxygen making up 28% and 12% of costs, respectively. For severe patients, staffing is the most important category of cost (43%). Oxygen and pharmaceuticals make up 36% and 13% of costs for severe patients, respectively.

In Kenya, the cost per patient day for EECC is estimated to be 2 USD, 14 USD and 37 USD for patients with moderate, severe and critical disease, respectively. The cost distributions are similar to those found in Tanzania, with staff time representing 88% of total costs for moderate patients. However, staff time is more expensive in Kenya than Tanzania. This results in staff costs being 54% and 36% of the costs for severe and critical patients, respectively, with other costs such as oxygen being a relatively less important contributor to overall costs (32% and 53% for severe and critical patients respectively). In contrast, the cost of pharmaceuticals is less in Kenya than in Tanzania at 0.54USD and 0.72USD per patient day for severe and critical patients, respectively. The costs per patient day of ACC in Kenya are 17 USD and 345 USD for severe and critical patients, respectively.

### Scenario analyses

In the scenario analyses, the cost of EECC ranges from 0.36 USD to 2.28 USD, 8.74 USD to 22.12 USD and 27.92 USD to 73.92 USD per patient day for patients in Tanzania with moderate, severe and critical COVID-19 respectively. For Kenya, the equivalent ranges are 1.39 USD to 2.75 USD, 11.68 USD–21.10 USD and 29.15 USD–50.91 USD. The costs of EECC were most sensitive to changes in the staff inputs across all three categories in both countries. The cost of oxygen provision had a relatively smaller effect on overall cost per patient day than changes in staff costs. The overall cost of care for moderate patients was more sensitive to changes in the input variables than the costs of severe and critical patients.

**Fig. 2:**
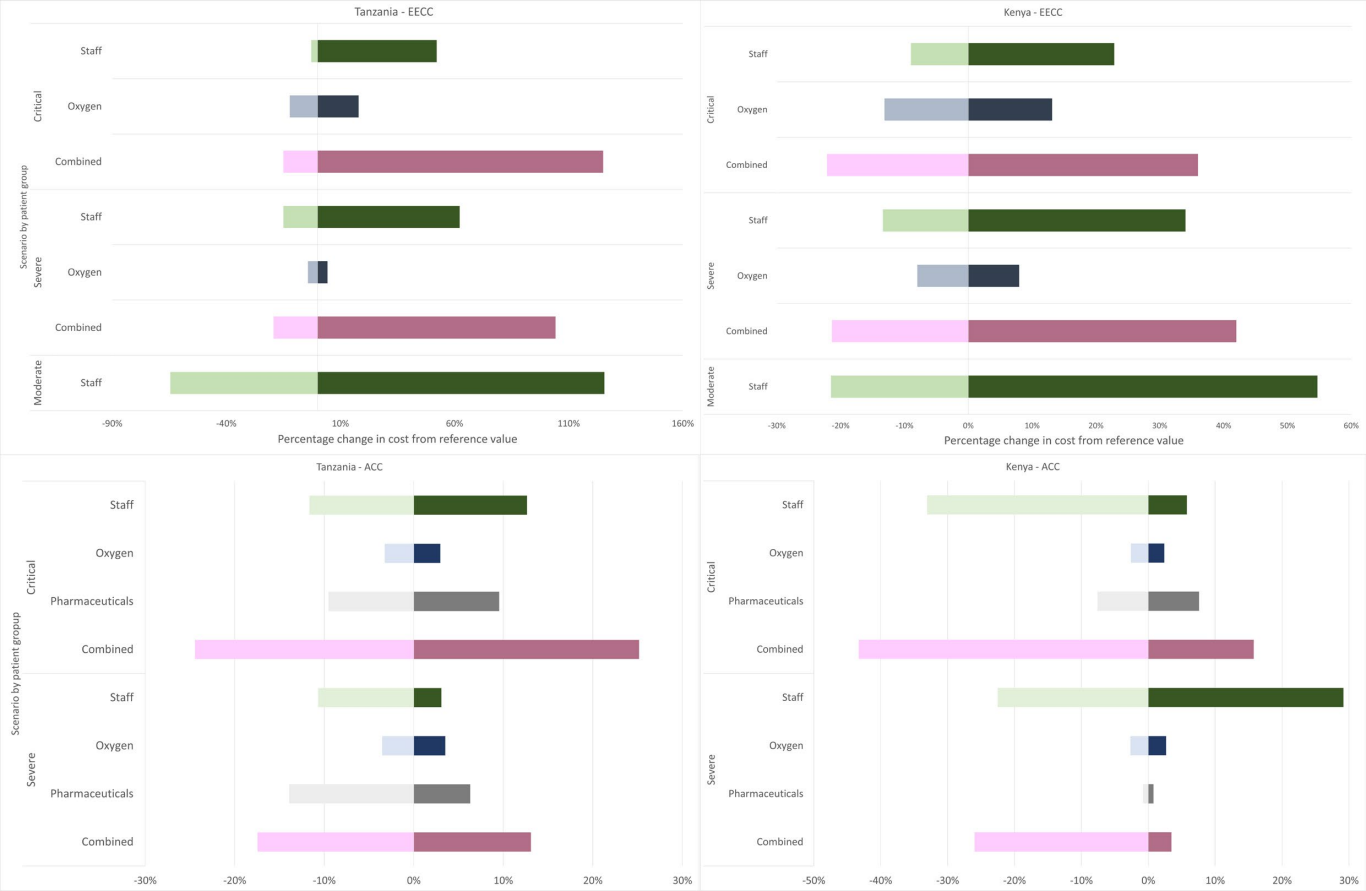
Scenario analyses

The cost per patient day of ACC ranged from 10.82 USD to 14.82 USD for severe patients and 224 USD to 372 USD for critical patients in Tanzania; and 12.83 USD–17.93 USD and 210 USD–428 USD, respectively, in Kenya. The costs were most sensitive to the assumptions relating to staff inputs (see Fig. 2) than to changes in the assumptions around oxygen costs.

## Discussion

We estimate that EECC costs 10.83/32.84 USD per day for a patient in Tanzania with severe/critical COVID-19 respectively and 14.86/37.43 USD per day in Kenya. Combining the results to estimate a cost of EECC in critical illness (assuming 74% of the critically ill have severe COVID-19 and 26% have critical COVID-19 [42]) gives approximate overall cost figures of 17 USD per patient per day for Tanzania and 21 USD for Kenya.

EECC costs reflect the essential needs of a critically ill patient and cover practices that are accepted as minimum standards of care to prevent and treat the deterioration of patients. The normative approach used in the costing allows us to examine what it would cost to provide EECC per patient over and above the other care provided in a hospital setting. We found that advanced critical care (ACC) is nearly 10 times more expensive per patient day than EECC in both Tanzania and Kenya.

Data on the effectiveness of critical care is generally poor. However, the ACC approach that was promoted early in the pandemic was often associated with poor outcomes [43]. At the same time, within a single funding pool, choosing to prioritise ACC requires a significant commitment of funding to a small group of patients. The low costs and lower tech approach inherent in delivering EECC suggest that EECC could be provided to many and indicate a need to prioritise EECC over ACC when resources are limited.

Strategies to scale up critical care in LMICs will depend on existing capacity. Distribution of critical care facilities that provide ACC type care in Tanzania are unequal [21]. And the same is true of Kenya [16]. Building capacity will take time and specialist resources that are not readily available. As EECC does not require these specialist skills, equipment and other resources, it is within reach and likely to represent a small portion of recurrent budgets of district hospital facilities (estimates of the average cost per inpatient day range from 17 to 71 USD in Tanzania and estimated to be 57 USD per inpatient day in Kenya (https://www.healthdata.org/sites/default/files/files/policy_report/2015/ABCE_Kenya_finalreport_Jan2015.pdf)) [24, 44]. ACC care in ICUs for all who would benefit may be a goal for all health systems. At the same time, for ACC to be an effective approach, ACC should be built on solid foundations of good quality EECC i.e. ensuring the basics are in place before more advanced approaches are attempted. In addition, if all patients had access to high quality EECC, this would minimize the risk of unnecessary deterioration to a state needing high-cost care in an ICU, as well as the associated higher risk of disability and death.

The breakdown of costs of EECC varies depending on the patient’s illness severity and is also likely to vary depending on the underlying diagnosis. For patients with severe or critical COVID-19, oxygen costs are between 30 and 60% of total costs per patient day. This importance of the availability of oxygen at all levels of the hospital has been echoed throughout the pandemic [45]. However, significance of oxygen in the EECC cost structure is in part due to the choice of COVID-19 as a tracer condition and is unlikely to be the same for critically ill patients with conditions that are not primarily associated with respiratory failure, such as post-operative care or sepsis. The relative importance of pharmaceuticals and staff time for patients with severe and critical COVID-19 varies between Kenya and Tanzania and is driven by the variation in human resource and pharmaceutical prices found across the two countries, as input quantities are fixed across the countries. The hotel costs associated with critical care and their contribution to overall costs varied across the countries due to the difference in hospital charge rates.

The cost estimates should be considered within the context of uncertainty and the normative approach taken. Clinical expertise was used to estimate resource use per patient day. While some data is available on COVID-19 resource use, full provision of EECC is currently a hypothetical scenario and so clinical expertise was the only available source. This was also a challenge for the ACC costing for critical patients for which pharmaceutical treatment can be complex and heterogenous. However, the impact of pharmaceutical costs on the comparison between ACC and EECC was marginal. Even when pharmaceutical costs are set to zero the ACC costs are over 5 times that of EECC for critical patients. Oxygen costs were also subject to some uncertainty given the volatility of the market during the pandemic. For this reason, oxygen costs were used from an established pre-pandemic international source. At the same time, the overall cost estimates were robust to changes in the oxygen costs. A final limitation relates to the cost per admission, which is derived from lengths of stay for COVID-19 patients from patients in the UK in the early stages of the pandemic, the best available data at the time of analysis [46].

EECC is a new concept, with the consensus on its construct developed as part of our research programme. As a result, no other costing of EECC has been carried out and there are no comparative cost data to compare and validate the results. Several studies have looked at overall COVID-19 care costs. Estimates for all the hospital care provided to critical patients were found to be 505 USD per day in Ethiopia, 599 USD per day in Kenya and, in South Africa, ranged between 62 and 79 USD per day for general ward based care to between 271 and 306 USD per day for ICU admissions [22, 32, 43, 47]. These costings do not distinguish between critical care and “other” care and therefore largely relate to diagnostics and inpatient day costs which are not included in our critical care costings.

The difference in EECC and ACC costs is most striking when comparing care for patients with critical COVID-19—ACC costs are 9 and 7 times higher EECC costs in Tanzania and Kenya, respectively. This difference is driven by the additional staffing, pharmaceutical and non-pharmaceutical consumables that are required by an ACC model of care. In addition to the delivery costs presented here, the infrastructure development to enable ACC care requires further investment in specialized human resources and equipment. A full economic evaluation would help answer the question of the most cost-effective approach. However, given the relative costs of delivery and investment, the likely marginal gains at the population level from investing in EECC to ensure all hospitals can provide these essential elements of critical care is likely to be more cost-effective than focused investment in ACC for a small number of hospital beds within the health system infrastructure.

## Conclusion

Essential Emergency and Critical Care (EECC) is a low-tech approach to all critically ill patients, including 40 care-processes that are effective, lifesaving and feasible. EECC is low-cost relative to more advanced critical and ICU care approaches (ACC) to caring for critically ill patients. The low costs suggest that EECC could be provided to many patients and that implementation of EECC should be prioritized over ACC when resources are limited.

## Supplementary Information

**Additional file 1.** The hospital resources required for providing (A) EECC and (B) ACC and (C) the subset of these resources that are included in the costing.

**Additional file 2.** Unit prices and sources of the unit prices.

**Additional file 3.** Scenarios used in PATH costing tool.

## Data Availability

The datasets used and analysed during the current study are available from the corresponding author on reasonable request.

## Abbreviations

ACC: Advanced critical care
EECC: Essential emergency critical care
ICU: Intensive care unit
LMIC: Low-and middle-income countries
PPE: Personal protective equipment
USD: United States dollars
