# Supplementary figures and images for: Essential emergency and critical care as a health system response to critical illness and the COVID19 pandemic: what does it cost?
Source: Cost Eff Resour Alloc. 2023 Feb 13;21:15. doi: 10.1186/s12962-023-00425-z (PMC9923646; doi:10.1186/s12962-023-00425-z)

# Additional file 2 – Unit prices and sources of the unit prices


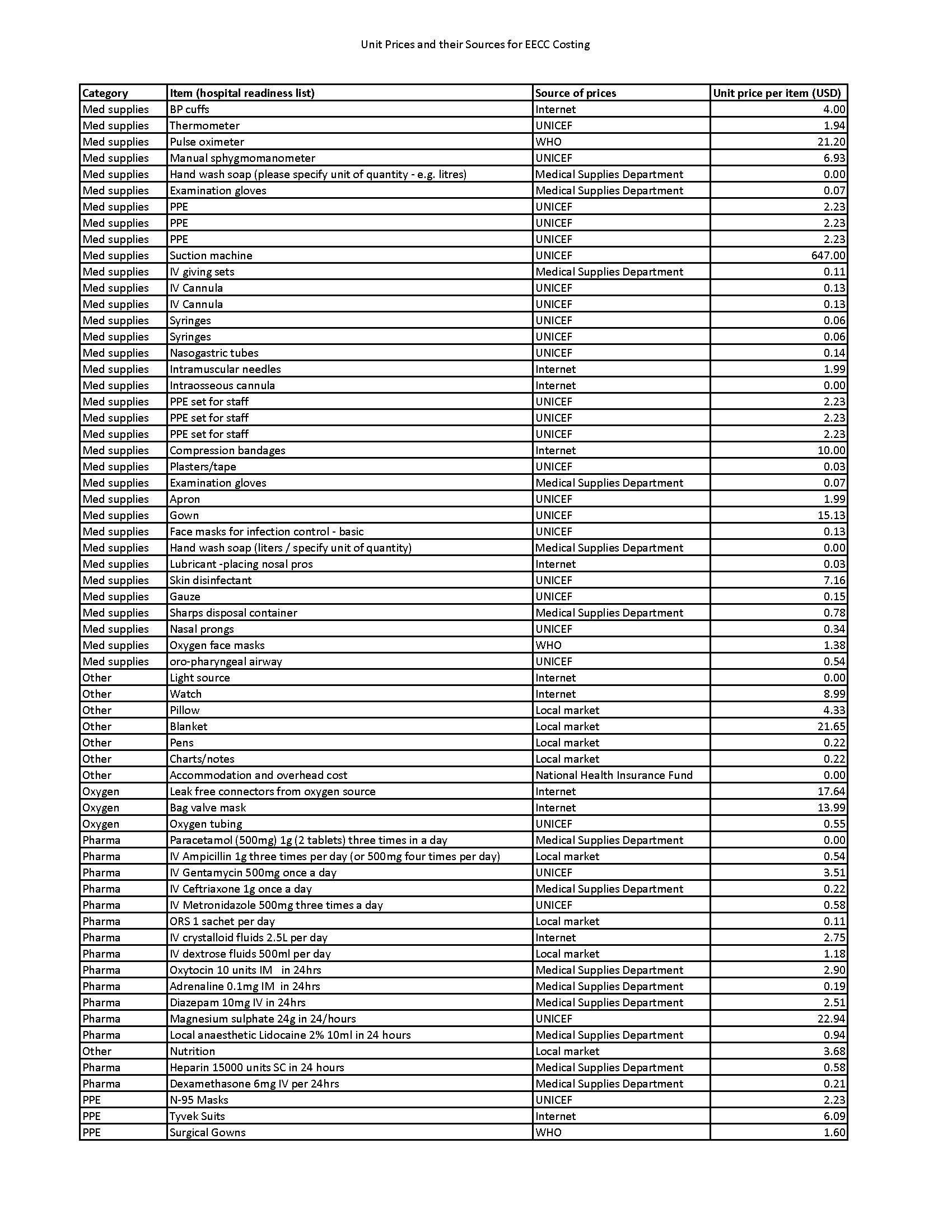

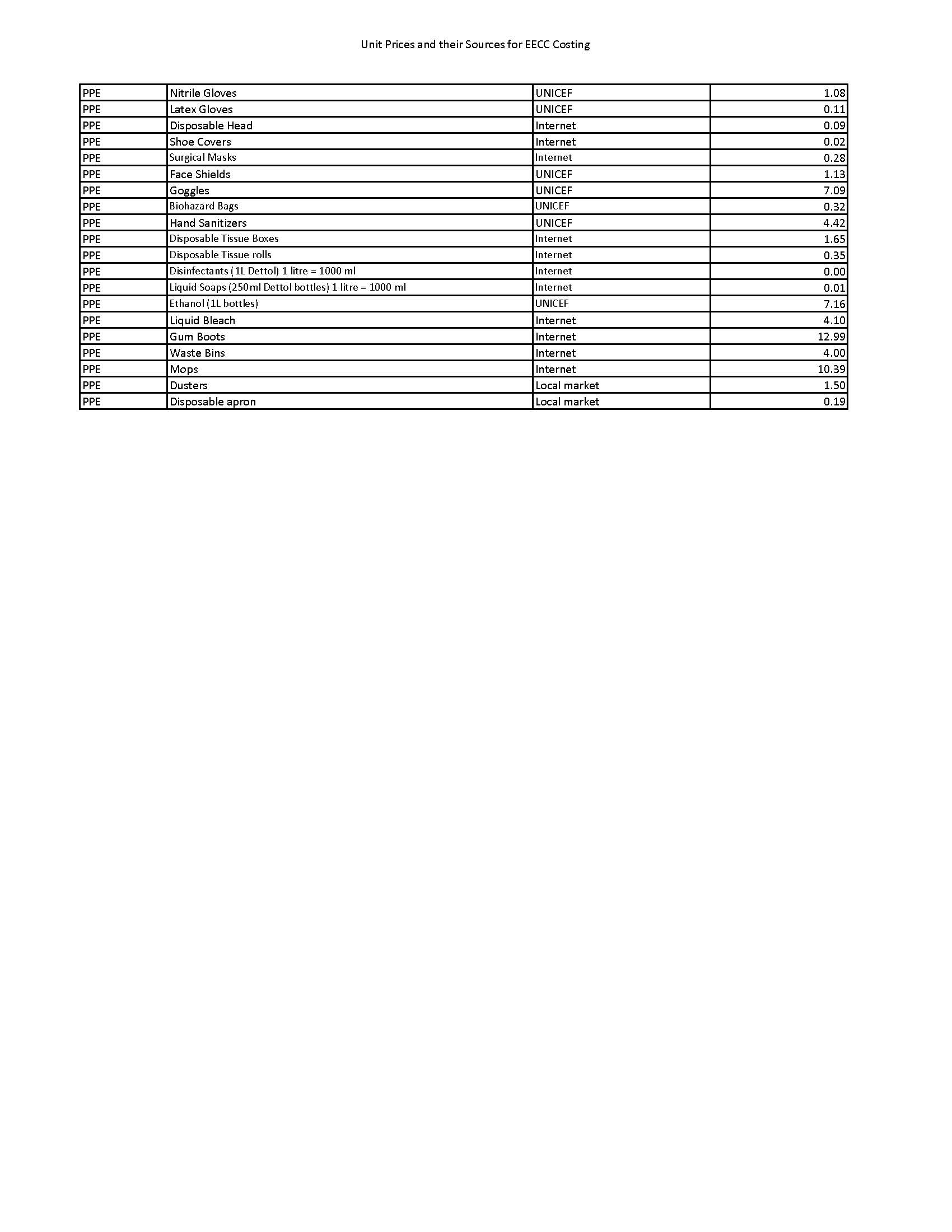


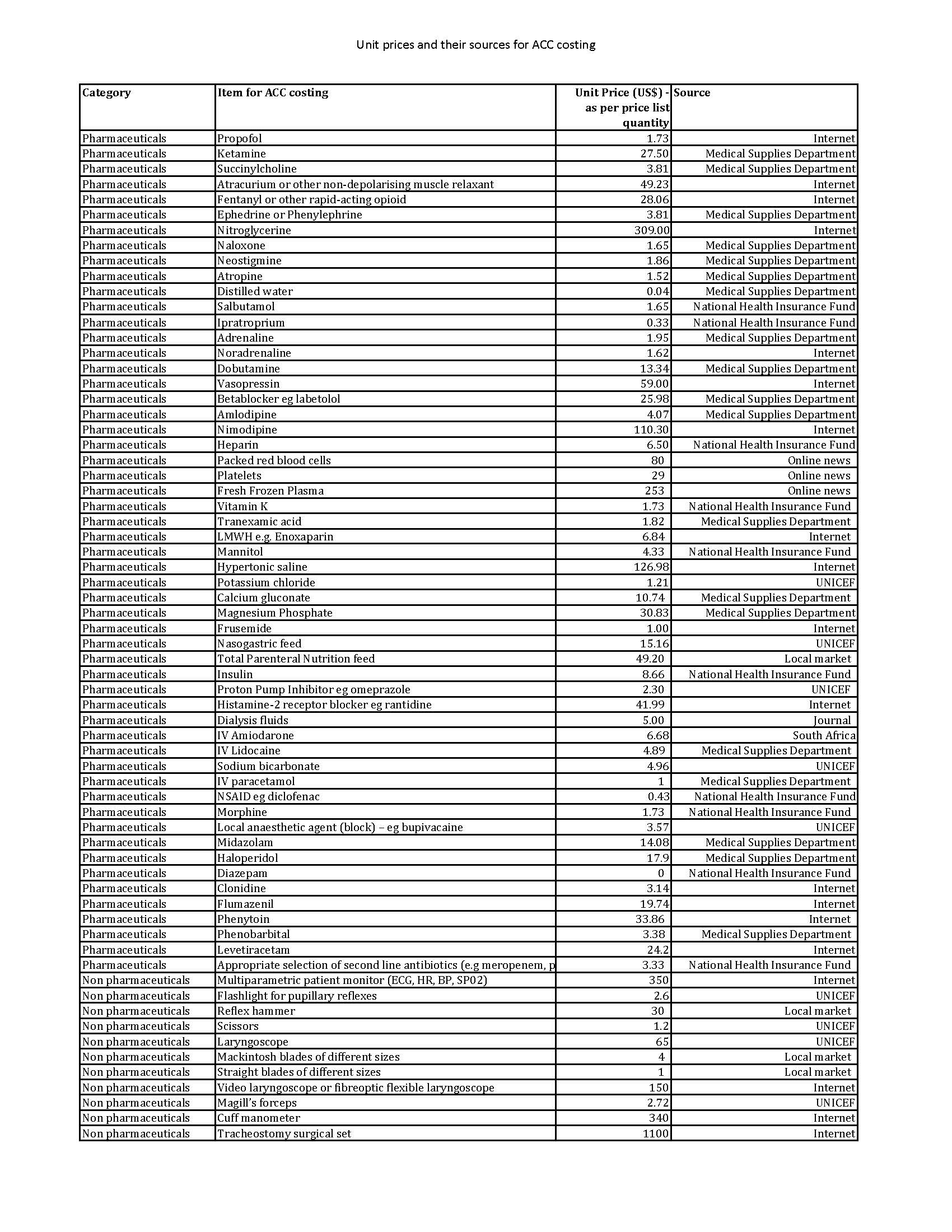

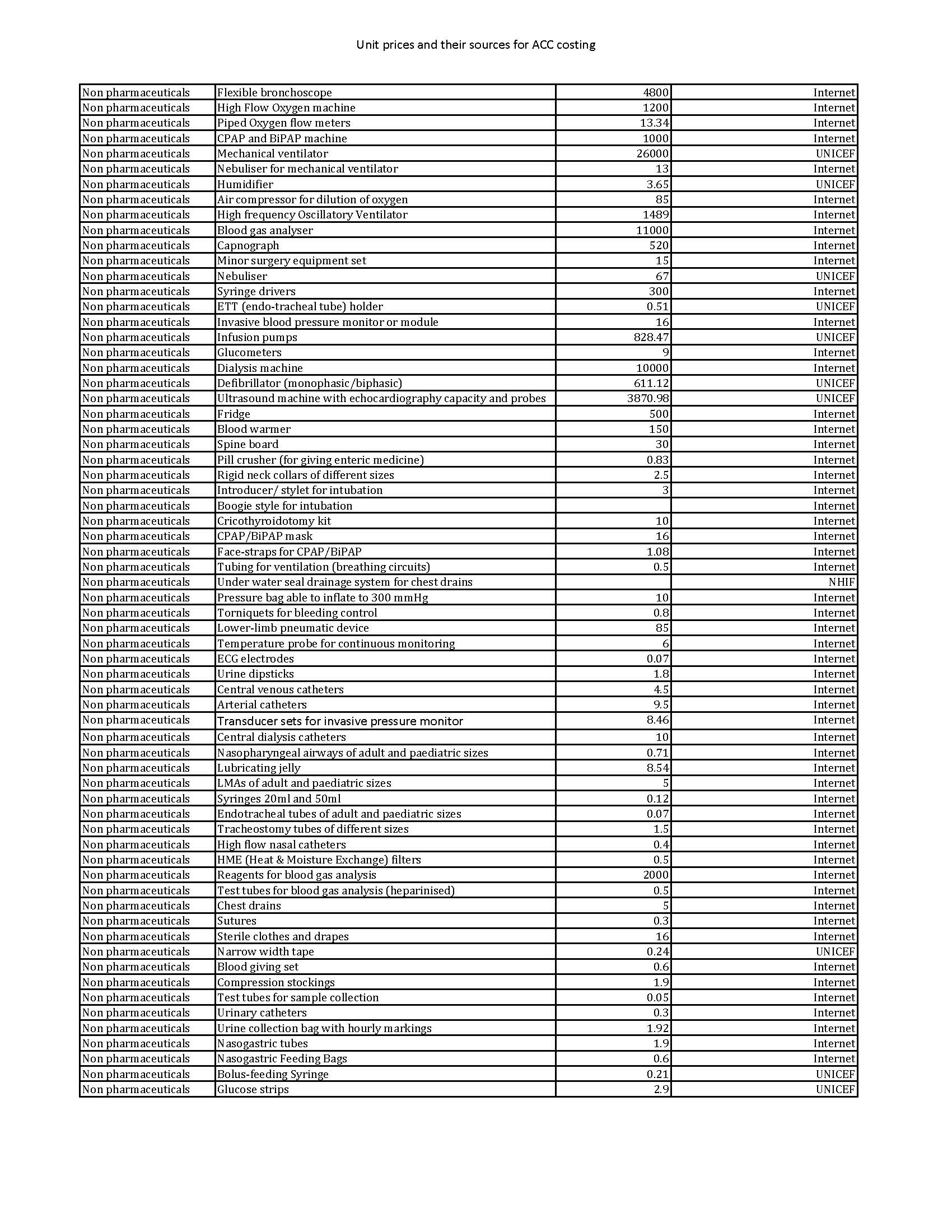

Supplement: Supplementary file 2 — Additional file 2. Unit prices and sources of the unit prices. [file 12962_2023_425_MOESM2_ESM.docx]

# Additional file 3

Scenarios used in PATH costing tool


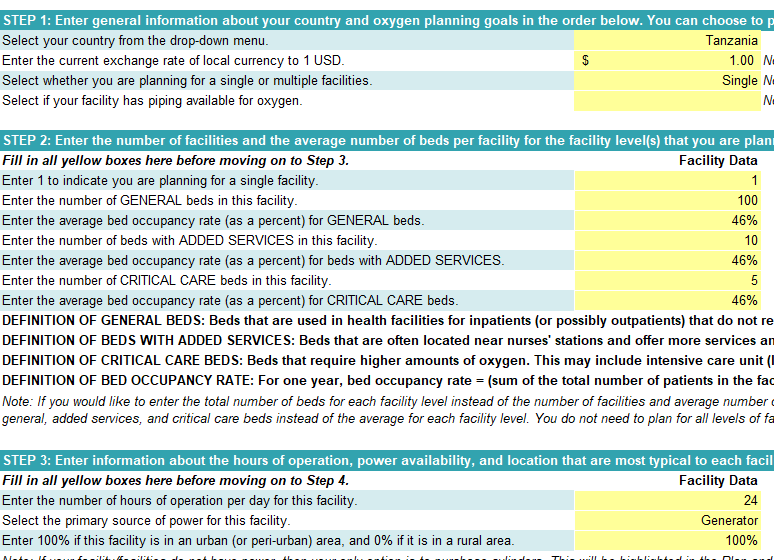


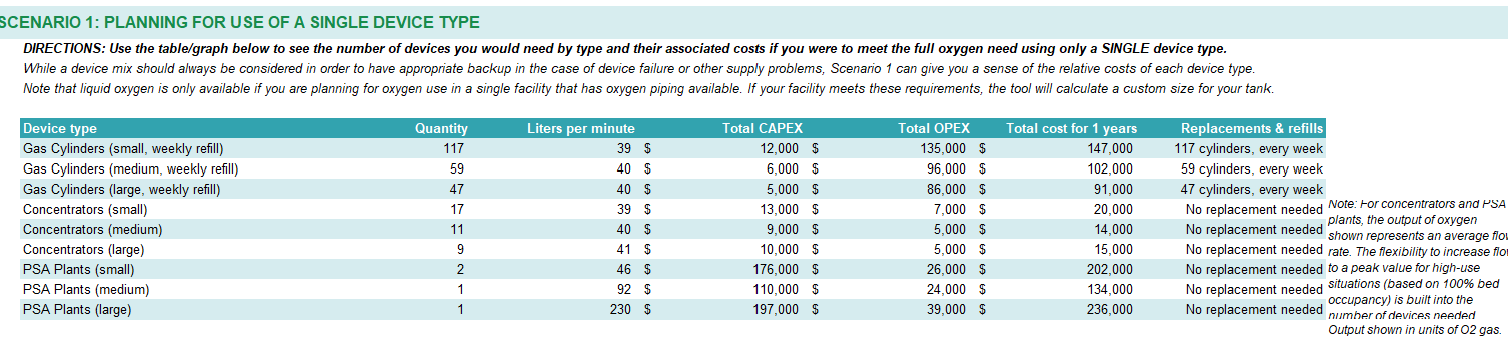

Supplement: Supplementary file 3 — Additional file 3. Scenarios used in PATH costing tool. [file 12962_2023_425_MOESM3_ESM.docx]
